# Comparison of national antimicrobial treatment guidelines, African Union

**DOI:** 10.2471/BLT.21.286689

**Published:** 2021-11-26

**Authors:** Jessica Craig, Kayli Hiban, Isabel Frost, Geetanjali Kapoor, Yewande Alimi, Jay K Varma

**Affiliations:** a Center for Disease Dynamics, Economics & Policy, 5636 Connecticut Ave NW, Washington, DC 20015, United States of America.; b Center for Disease Dynamics, Economics & Policy, New Delhi, India.; c Africa Centres for Disease Control and Prevention, Addis Ababa, Ethiopia.

## Abstract

**Objective:**

To identify and compare antimicrobial treatment guidelines from African Union (AU) Member States.

**Methods:**

We reviewed national government agency and public health institutes’ websites and communicated with country or regional focal points to identify existing treatment guidelines from AU Member States. We included guidelines if they contained disease-, syndrome- or pathogen-specific treatment recommendations and if those recommendations included antimicrobial name or class, dosage and therapy duration. The scope of the review was limited to infections and clinical syndromes that often have a bacterial cause. We assessed treatment guidelines for alignment with the Grading of Recommendations Assessment, Development and Evaluation (GRADE) criteria. We compared treatment recommendations for various common bacterial infections or clinical syndromes described across national guidelines and those described in three World Health Organization guidelines.

**Findings:**

We identified 31 treatment guidelines from 20 of the 55 (36%) AU Member States; several countries had more than one treatment guideline that met our inclusion criteria. Fifteen (48%) guidelines from 10 countries have been published or updated since 2015. Methods used to develop the guidelines were not well described. No guidelines were developed according to the GRADE approach. Antimicrobial selection, dosage and duration of recommended therapies varied widely across guidelines for all infections and syndromes.

**Conclusion:**

AU Member States lack antimicrobial treatment guidelines that meet internationally accepted methods and that draw from local evidence about disease burden and antimicrobial susceptibility.

## Résumé

**Objectif:**

Identifier et comparer les directives de traitement antimicrobien des États Membres de l'Union africaine (UA).

**Méthodes:**

Nous avons examiné les sites Internet de l'agence gouvernementale nationale et des organismes de santé publique, mais aussi contacté les points focaux nationaux et régionaux afin d'identifier les directives de traitement existant au sein des États Membres de l'UA. Nous avons inclus toute directive incluant des recommandations de prise en charge spécifique des maladies, syndromes ou agents pathogènes, et mentionnant le nom ou la catégorie d'antimicrobiens, le dosage ainsi que la durée du traitement. La portée de notre revue s'est limitée aux infections et syndromes cliniques fréquemment causés par des bactéries. Nous avons ensuite évalué ces directives de traitement afin de déterminer leur correspondance avec les critères du système GRADE (Grading of Recommendations, Assessment, Development and Evaluation, soit «grade donné aux recommandations, examen, élaboration et évaluation»). Enfin, nous avons comparé les recommandations de traitement pour plusieurs infections bactériennes ou syndromes cliniques courants reprises dans les différentes directives nationales avec celles décrites dans trois des lignes directrices de l'Organisation mondiale de la Santé.

**Résultats:**

Nous avons identifié 31 directives de traitement dans 20 des 55 (36%) États Membres de l'UA; de nombreux pays possédaient plus d'une directive répondant à nos critères d'inclusion. Quinze (48%) directives issues de 10 pays ont publiées ou mises à jour depuis 2015. Les méthodes employées pour les développer n'étaient pas bien documentées. Aucune directive n'avait été élaborée selon l'approche GRADE. Le choix des antimicrobiens, leur dosage et la durée de traitement conseillée variaient énormément d'une directive à l'autre, pour l'ensemble des syndromes et infections.

**Conclusion:**

Les États Membres de l'UA manquent de directives de traitement antimicrobien conformes aux méthodes universellement reconnues, et qui s'inspirent des données locales relatives à la charge de morbidité et à la sensibilité aux antimicrobiens.

## Resumen

**Objetivo:**

Identificar y comparar las directrices de tratamiento antibiótico de los Estados miembros de la Unión Africana (UA).

**Métodos:**

Se revisaron los sitios web de las agencias gubernamentales nacionales y de los institutos de salud pública y se consultó a los centros de coordinación nacionales o regionales para identificar las directrices de tratamiento existentes en los Estados miembros de la UA. Se incluyeron las directrices si contenían recomendaciones de tratamiento específicas para la enfermedad, el síndrome o el patógeno y si esas recomendaciones incluían el nombre o la clase de antibiótico, la dosis y la duración del tratamiento. El alcance de la revisión se limitó a las infecciones y los síndromes clínicos que suelen tener una causa bacteriana. Se evaluó la alineación de las directrices de tratamiento con los criterios de Evaluación, Desarrollo y Valoración de Grados de Recomendación (GRADE). Se compararon las recomendaciones de tratamiento para diversas infecciones bacterianas comunes o síndromes clínicos descritos en las directrices nacionales y las descritas en tres directrices de la Organización Mundial de la Salud.

**Resultados:**

Se identificaron 31 directrices de tratamiento de 20 de los 55 (36 %) Estados miembros de la UA; varios países tenían más de una directriz de tratamiento que cumplía nuestros criterios de inclusión. Quince (48 %) directrices de 10 países se han publicado o actualizado desde 2015. Los métodos utilizados para elaborar las directrices no estaban bien descritos. Ninguna de las directrices se elaboró según el enfoque GRADE. La selección de los antibióticos, la dosis y la duración de los tratamientos recomendados variaron en gran medida entre las directrices para todas las infecciones y síndromes.

**Conclusión:**

Los Estados miembros de la UA carecen de directrices de tratamiento antibiótico que cumplan con los métodos aceptados a nivel internacional y que se basen en las evidencias locales sobre la carga de las enfermedades y la sensibilidad a los antibióticos.

## ملخص

الغرض تحديد المبادئ التوجيهية للعلاج بمضادات الميكروبات، ومقارنتها مع الدول الأعضاء في الاتحاد الأفريقي (AU).

الطريقة قمنا بمراجعة المواقع الإلكترونية للوكالة الحكومية الوطنية، ومعاهد الصحة العامة، وتواصلنا مع النقاط المركزية القطرية أو الإقليمية لتحديد المبادئ التوجيهية الحالية للعلاج من الدول الأعضاء في الاتحاد الأفريقي. قمنا بتضمين المبادئ التوجيهية إذا كانت تحتوي على توصيات للعلاج خاصة بالمرض، أو بالمتلازمة، أو بالعوامل المسببة للمرض، وإذا تضمنت تلك التوصيات اسم أو فئة مضادات الميكروبات، والجرعة، ومدة العلاج. اقتصر نطاق المراجعة على أنواع العدوى والمتلازمات الإكلينيكية التي غالبًا ما يكون لها سبب بكتيري. وقمنا بتقييم المبادئ التوجيهية للعلاج من حيث توافقها مع معايير درجات تقييم التوصيات وتطويرها وتقييمها (GRADE). قمنا بمقارنة توصيات العلاج لمختلف أنواع العدوى البكتيرية الشائعة، أو المتلازمات الإكلينيكية الواردة في المبادئ التوجيهية الوطنية، وتلك الموضحة في المبادئ التوجيهية الثلاث لمنظمة الصحة العالمية.

النتائج قمنا بتحديد 31 من المبادئ التوجيهية للعلاج من 20 دولة من إجمالي 55 دولة (36%) من الدول الأعضاء في الاتحاد الأفريقي؛ وكان لدى العديد من الدول أكثر من مبدأ توجيهي واحد يفي بمعايير التضمين لدينا. تم نشر أو تحديث خمسة عشر (48%) من المبادئ التوجيهية من 10 دول منذ عام 2015. لم يتم وصف الطرق المستخدمة لتطوير المبادئ التوجيهية بشكل جيد. لم يتم تطوير أية مبادئ توجيهية وفقًا لأسلوب GRADE. اختلف اختيار مضادات الميكروبات وجرعتها ومدة العلاجات الموصى بها على نطاق واسع عبر المبادئ التوجيهية لجميع أنواع العدوى والمتلازمات.

الاستنتاج تفتقر الدول الأعضاء في الاتحاد الأفريقي إلى المبادئ التوجيهية للعلاج بمضادات الميكروبات التي تفي بالأساليب المقبولة دوليًا، والتي تعتمد على الأدلة المحلية حول عبء المرض وقابلية التأثر بمضادات الميكروبات.

## 摘要

**目的:**

旨在明确和比较非洲联盟 (AU) 成员国的抗菌药物指南。

**方法:**

我们审查了国家政府机关和公共卫生机构的网站并与国家或地方性的联络点取得了沟通，以确定非洲联盟成员国的当前药物指南。如果指南包含与具体疾病、综合征或病原体有关的药物推荐，而且这些推荐中提及了抗菌药物的名称或级别、剂量和治疗周期，那么我们将这些指南纳入我们的研究中。审查的范围限于通常由细菌引起的感染和临床症状。我们根据推荐、评估、发展和评估等级 (GRADE) 标准评估了药物指南。我们针对国家指南和世界卫生组织指南中描述的各种常见细菌感染或临床症状对不同治疗推荐进行了比较。

**结果:**

我们确定了来自 55 个非洲联盟成员国中的 20 个国家 (36%) 的 31 项治疗指南；有几个国家的多项治疗指南符合我们的纳入标准。自 2015 年以来，来自 10 个国家的十五项 (48%) 指南已发布或更新。。但并未清楚描述制定指南所采用的方法，也没有指南是依据 GRADE 方法制定的。不同指南针对各种感染和综合征推荐的抗菌药物选择、剂量和持续时间差异很大。

**结论:**

非洲联盟成员国缺乏按照国际公认的方法且借鉴关于疾病负担和抗菌药物耐药性的地方性证据制定的抗菌药物治疗指南。

## Резюме

**Цель:**

Определить и сравнить руководства по применению противомикробных препаратов в государствах-членах Африканского союза (АС).

**Методы:**

Авторы изучили веб-сайты национальных правительственных ведомств и институтов общественного здравоохранения и связались с национальными или региональными координаторами, чтобы определить существующие руководства по применению противомикробных препаратов в государствах-членах Африканского союза. К рассмотрению допускались руководства, содержавшие рекомендации по лечению конкретных заболеваний, синдромов или патогенов, включавшие название или класс противомикробного препарата, дозировку и продолжительность терапии. Сфера охвата обзора ограничивалась инфекциями и клиническими синдромами, которые часто имеют бактериальное происхождение. Авторы оценили руководства по лечению на предмет их соответствия критериям Системы классификации, оценки, разработки и экспертизы рекомендаций (GRADE). Авторы сравнили рекомендации по лечению различных распространенных бактериальных инфекций или клинических синдромов, описанных в национальных руководствах и в трех руководствах Всемирной организации здравоохранения.

**Результаты:**

Было рассмотрено 31 руководство по лечению в 20 государствах-членах АС из 55 (36%); в нескольких странах имелось несколько руководств по лечению, соответствующих критериям включения. Пятнадцать руководств (48%) из 10 стран были опубликованы или обновлены начиная с 2015 года. Методы, использованные для разработки руководств, описаны недостаточно хорошо. Никаких руководств в соответствии с подходом GRADE разработано не было. Выбор противомикробных препаратов, дозировка и продолжительность рекомендованной терапии для всех инфекций и синдромов существенно различались в руководствах.

**Вывод:**

В государствах-членах АС отсутствуют руководства по применению противомикробных препаратов, которые соответствовали бы международно признанным методам и основывались на местных данных о бремени болезни и чувствительности к противомикробным препаратам.

## Introduction

Antimicrobial resistance poses significant public health challenges and threatens the ability to treat many common infectious diseases. Across Africa, rising rates of drug resistance have been documented for the pathogens that cause tuberculosis, pneumonia, diarrhoeal diseases, malaria and sexually transmitted infections.^1^ Documented levels of antimicrobial resistance rates are likely lower than the actual level due to limited surveillance activities and laboratory capacity and lack of consistent access and utilization of antimicrobial susceptibility testing.^2^

Major drivers of antimicrobial resistance include: the misuse and overuse of antimicrobials in the human health and agricultural sectors; lack of access to clinically appropriate antimicrobials; lack of regulation and/or regulatory enforcement restricting access to antimicrobials to prescription-only use; and a higher burden of infectious diseases which is driven by low vaccination coverage and limited water, sanitation and hygiene infrastructure.^2^^–^^4^ Given these risk factors for antimicrobial resistance, low- and middle-income countries are at a higher risk for the propagation of resistant pathogens compared to high-income countries. Moreover, the public health consequences of antimicrobial resistance may be higher in African countries because of failure to diagnose antimicrobial-resistant infections and the absence of expensive second-line therapies.

In 2018, the Africa Centres for Disease Control and Prevention (Africa CDC) released its Framework for Antimicrobial Resistance, a strategy to improve surveillance, delay emergence, limit transmission and mitigate harm from antimicrobial-resistant pathogens in Africa.^5^ African Union (AU) Member States and stakeholders identified priority activities for implementing Africa CDC’s framework. To delay the emergence and mitigate harm of antimicrobial resistance, many expert organizations recommended that antimicrobials only be used in accordance with established clinical guidelines that outline when, what and how to prescribe. However, representatives of the AU Member States noted that, except for select diseases such as human immunodeficiency virus (HIV) infection, tuberculosis and malaria, many health-care providers do not have country-specific guidelines and must rely on individual judgement or treatment guidelines developed outside of Africa.

Standardized treatment guidelines are an important tool to ensure the appropriate and optimal use of antimicrobials in the human health sector, particularly when informed by local data and combined with antimicrobial stewardship programmes.^6^^,^^7^ We sought to identify existing standardized treatment guidelines from African government agencies or public health institutes, to assess their completeness and quality, and compare the antimicrobial treatment therapies described across the guidelines.

## Methods

We reviewed the websites of health ministries, national public health institutes or equivalent national government agencies of all 55 AU Member States for relevant published standardized treatment guidelines.^8^ We conducted two reviews, the first between December 2018 and March 2019 and the second between May and June 2021. For countries without functioning health ministry websites or for countries where guidelines were not readily or apparently available, we contacted in-country Africa CDC focal points by email to help identify existing standardized treatment guidelines. In-country focal points were identified through Africa CDC Regional Collaborating Centres and included regional World Health Organization (WHO) staff. Two subsequent emails were sent to each focal person if immediate response did not occur. We were unable to identify specific in-country focal points in Djibouti, Libya, and Somalia; a regional focal point was contacted in these cases. In total, 15 national or regional focal points were contacted with two not responding.

To inform our work, we presented our proposed methods and inclusion criteria to a panel of 28 infectious disease clinicians and public health experts representing 13 AU Member States. This consultation resulted in exclusion of national and regional guidelines developed by professional bodies and organizations, given that these guidelines may not be widely or systematically available or used in health facilities across the continent. An evaluation of the access and utilization of guidelines developed by these bodies would be required to determine if they are consistently used by clinicians treating infectious diseases. Furthermore, these guidelines may not represent feasible clinical options at the national or continental level given challenges in access to various antimicrobials or other diagnostic or treatment paradigms.

We limited the scope of the review to bacterial infections and clinical syndromes that often have a bacterial cause. Disease-specific guidelines for HIV, malaria, tuberculosis and other infections or syndromes addressed by national or vertical disease control programmes in Africa were excluded. We considered guidelines published in any major continental language. For final review, we included guidelines only if they contained disease-, syndrome- or pathogen-specific treatment recommendations and recommendations included specific name or class of antimicrobial, dosage and duration of therapy.

Standardized treatment guidelines that met the inclusion criteria were assessed for alignment with the Grading of Recommendations Assessment, Development and Evaluation (GRADE) criteria.^9^ This included a review of information describing the methods used in guidelines development including whether the guidelines underwent external technical review or were informed by a systematic review of local, national or regional antimicrobial resistance or disease burden data.^9^

We compiled antimicrobial treatment recommendations provided in each guideline for common bacterial diseases, including information on drug (or class of antimicrobial) selection, dosage and duration of therapy. Recommendations were segregated by adult, paediatric and/or neonatal patient populations as specified in the source guidelines. Special populations (e.g. pregnant women, patients with penicillin allergies) were included if noted in the source guidelines. Alternative and second-line therapy recommendations were also recorded. We did not extract other clinical information, such as case definition, recommended diagnostic testing and non-antimicrobial therapy treatments (e.g. vitamin supplements or pain management).

For comparison regarding first- and second-line antimicrobial selection, we also consulted the 2019 *WHO Model list of essential medicines*,^10^ the 2019 *WHO* *Model list of essential medicines for Children*^11^ and the *Pocket book of hospital care for children*, 2nd edition.^12^ The WHO model lists only provided drug selection recommendations and did not provide recommended treatment dosages or durations; therefore, further comparison of recommended therapy was not possible. Finally, we compared treatment regimens provided across individual guidelines, including the WHO model lists, to assess variability in antimicrobial treatment recommendations.

## Results

### Description of guidelines

Table 1 presents the 31 standardized treatment guidelines from 20 countries that met the inclusion criteria.^13^^–^^43^ Several countries had more than one published guideline included for final review: Ethiopia (2 guidelines), Kenya (2), the Seychelles (2), South Africa (5), Tunisia (3), Uganda (2) and Zambia (2). Despite extensive efforts, we did not identify existing standardized treatment guidelines for the remaining 35 AU Member States. Year of publication or last update ranged from 2001 to 2018; two standardized treatment guidelines did not state the year of publication. Only 15 (48%) of 31 standardized treatment guidelines from 10 countries have been published or updated since 2015. Four guidelines were published in French and the remaining were published in English. All guidelines were published by the health ministry or other national health agencies or research institutes. Twenty-three (74%) guidelines provided treatment recommendations for both adult and paediatric patient populations, five provided only adult-specific guidelines and three provided paediatric-only recommendations. Overall, the description methods used in the development of the guidelines was poor and no guidelines were developed according to the GRADE approach. Only three (10%) guidelines reported that the treatment recommendations considered available antimicrobial resistance data, while 10 (32%) guidelines stated that data on local disease burden was considered when developing recommendations.

**Table 1:** Summary of standard treatment guidelines included in the study on antimicrobial treatment recommendations, African Union

Country	Title	Publication year	Adult	Paediatric
Eswatini^13^	*Standard treatment guidelines and essential medicines list of common medical conditions in the Kingdom of Swaziland*	2012	Yes	Yes
Ethiopia^14^	*Guideline on cholera outbreak management*	2011	Yes	Yes
Ethiopia^15^	*National guidelines for the management of sexually transmitted infections using syndromic approach*	2015	Yes	Yes
Gambia^16^	*The Gambia standard drug treatment guide*	2001	Yes	Yes
Ghana^17^	*Standard treatment guidelines*	2010	Yes	Yes
Kenya^18^	*Clinical guidelines for the management and referral of common conditions at levels 4–6: hospitals*	2009	Yes	Yes
Kenya^19^	*Guidelines on cholera control*	2002	Yes	Yes
Liberia^20^	*2nd edition national standard therapeutic guidelines and essential medicines list Liberia, 2017*	2017	Yes	Yes
Malawi^21^	*Malawi standard treatment guidelines (MSTG)*	2015	Yes	Yes
Morocco^22^	*Directives de prise en charge de l'enfant malade de moins de cinq ans^a^*	2016	No	Yes
Namibia^23^	*Namibia standard treatment guidelines*	2011	Yes	Yes
Nigeria^24^	*Nigeria standard treatment guidelines*	2016	Yes	Yes
Rwanda^25^	*Internal medicine clinical treatment guidelines*	2012	Yes	Yes
Seychelles^26^	*Antimicrobial guidelines for management of infections in hospitals*	2018	Yes	Yes
Seychelles^27^	*Guidelines for antibiotic prescribing in the primary health care services*	2017	Yes	Yes
Somalia^28^	*Somali treatment guidelines in line with the essential package of health services, primary health unit STGs*	2015	Yes	Yes
South Africa^29^	*Standard treatment guidelines and essential medicines list for South Africa: hospital level paediatrics*	2017	No	Yes
South Africa^30^	*Standard treatment guidelines and essential medicines list for South Africa: hospital level adults*	2015	Yes	Yes
South Africa^31^	*Standard treatment guidelines and essential medicines list for South Africa: primary health care level*	2018	Yes	Yes
South Africa^32^	*Guidelines on leprosy control in South Africa*	2011	Yes	No
South Africa^33^	*Listeriosis: clinical recommendations for diagnosis and treatment*	2017	Yes	No
Sudan^34^	*Sudan national standard treatment guidelines*	2014	Yes	Yes
Tunisia^35^	*Antibiotherapie des infectious osteo-articulaires aigues communautaires a pyogenes – recommandations nationales fevrier 2006^a^*	2006	Yes	No
Tunisia^36^	*Antibiotherapie des pyelonephrities aigues communautaires de l'adulte^a^*	NA	Yes	No
Tunisia^37^	*L'Antibiotherapie dans les infections respiratoires basses acquises de l'adulte traité en ville^a^*	NA	Yes	No
Uganda^38^	*Prevention and control of cholera*	2017	Yes	Yes
Uganda^39^	*Uganda clinical guidelines 2016 *	2016	Yes	Yes
United Republic of Tanzania^40^	Standard treatment guidelines (STG) & the national essential medicines list for mainland Tanzania	2007	Yes	Yes
Zambia^41^	*Standard treatment guidelines, essential medicines list and essential laboratory supplies list for Zambia*	2013	Yes	Yes
Zambia^42^	*Essential newborn care guidelines*	2014	No	Yes
Zimbabwe^43^	*7th essential medicines list and standard treatment. Guidelines for Zimbabwe*	2015	Yes	Yes

NA: not available.

^a^ Guideline published in French.

We extracted and compiled treatment regimen recommendations for 27 infections and/or syndromes for adult patients and 26 for paediatric patients (Table 2). Meningitis, conjunctivitis, mild, moderate or non-severe pneumonia and urinary tract infection were the most common bacterial infections or clinical syndromes included in guidelines for adult patients; each were covered by 14 (70%) countries. Thirteen (65%) countries had guidelines that provided treatment recommendations for adult patients for acute otitis media, cholera, impetigo and pelvic inflammatory disease, while guidelines from 12 (60%) countries provided recommendations for acute and/or chronic bronchitis, syphilis and typhoid or enteric fever. Cholera and meningitis were the most frequently included infections or clinical syndromes for paediatric patients followed by acute otitis media, conjunctivitis, impetigo and typhoid or enteric fever. The WHO model list provided treatment regimen recommendations for adult populations for 48% (13/27) of the bacterial infections or syndromes commonly included in the standardized treatment guidelines of AU Member States. The WHO model list for children and the pocket book provided recommendations for 50% (13) and 27% (7), respectively, of the 26 infections or syndromes covered by the guidelines.

**Table 2:** Bacterial infections or syndromes covered by African standard treatment guidelines, *WHO Model list of essential medicines* and *Pocket book of hospital care for children*, 2001–2019

Infection or syndrome	With adult patient recommendations		With paediatric patient recommendations		
	No. of countries	Covered by WHO Model list^a^	No. of countries	Covered by WHO Model list for children^b^	Covered by the pocket book^c^
Meningitis	14	Yes	14	Yes	Yes
Non-severe pneumonia^d^	14	Yes	9	Yes	Yes
Urinary tract infection	14	Yes	7	Yes	Yes
Conjunctivitis	14	No	11	No	No
Cholera	13	Yes	14	Yes	No
Acute otitis media	13	Yes	13	Yes	No
Impetigo	13	No	11	No	No
Pelvic inflammatory disease	13	No	1	No	No
Typhoid or enteric fever	12	Yes	10	Yes	Yes
Syphilis	12	Yes	6	Yes	No
Acute and/or chronic bronchitis	12	No	4	No	No
Gonorrhoea or chlamydia	11	Yes	1	Yes	No
Dental abscess	10	Yes	7	Yes	No
Trichomoniasis and bacterial vaginosis	10	Yes	1	No	No
Cellulitis	10	No	9	No	No
Tonsilitis	10	No	8	No	No
Dysentery^e^	9	Yes	9	Yes	Yes
Chronic otitis media	9	Yes	7	Yes	No
Tetanus	9	No	9	No	No
Severe pneumonia	8	Yes	8	Yes	Yes
Sepsis or septicaemia	7	No	9	Yes	Yes
Gingivitis	7	No	6	No	No
Brucellosis	6	No	4	No	No
Diphtheria	6	No	3	No	No
Cutaneous anthrax	6	No	2	No	No
Peritonsillar abscess	6	No	0	No	No
Plague	5	No	4	No	No

WHO: World Health Organization.

^a^ 2019 *WHO Model list of essential medicines*.^10^

^b^ 2019 *WHO Model list of essential medicines for children*.^11^

^c^ *Pocket book of hospital care for children*.^12^

^d^ Includes also moderate or mild pneumonia.

^e^ Includes bacillary dysentery and shigellosis.

### Comparison of regimens

Antimicrobial selection and dosage and duration of recommended therapies varied across guidelines for all bacterial infections or clinical syndromes included in this analysis. Few guidelines provided organism-specific treatment recommendations or guidance on tailoring antimicrobial therapy according to bacterial culture or antimicrobial susceptibility testing results. Complete data sets for all infections and clinical syndromes are available in the authors’ data repository.^44^ For brevity, we present illustrative examples below showing the range of recommendations: a comparison of treatment recommendations for bacterial meningitis in paediatric and neonatal patient populations and for bronchitis in adult patients.

#### Paediatric population

A total of 14 countries provided recommendations for the treatment of acute bacterial or suspected bacterial meningitis in paediatric and/or neonatal patient populations (Table 3). Of those, eight (57%) countries provided organism-specific drug selection recommendations or provided organism-specific therapy durations whereas the other standardized treatment guidelines did not specify a causative agent or provided recommendations in situations when the causative agent was unknown. The WHO model list for children also provided treatment recommendations for acute bacterial meningitis with no organism-specific recommendations where the first-choice drug selections were cefotaxime for neonates and ceftriaxone for children. Second-choice antimicrobials included meropenem for neonates and amoxicillin, ampicillin, benzylpenicillin and chloramphenicol for children older than 2 years. Across the guidelines that provided treatment recommendations for bacterial meningitis in paediatric patients (excluding neonates) where the causative agent was not specified or in cases where it is unknown, recommended drug selections for monotherapy included benzylpenicillin, ampicillin, ceftriaxone and chloramphenicol. The recommended combination therapies were benzylpenicillin plus chloramphenicol. For neonates, recommended drug selections for monotherapy included ceftriaxone and fluconazole, and combination therapies included ampicillin IV plus gentamicin and benzylpenicillin plus chloramphenicol. Multiple guidelines recommended the same first-choice antimicrobial; however, dosage and therapy duration varied. For instance, dosage and duration recommendations for monotherapy treatment of paediatric patients (excluding neonates) with ceftriaxone ranged from 100 mg/kg daily for 7 days to 100 mg/kg daily for 10 to 14 days and 50–100 mg/kg every 12 hours for 14 days.

**Table 3:** Recommendations for the treatment of bacterial meningitis in neonatal and paediatric patients from a subset of African standard treatment guidelines, 2001–2019

Country	Recommended first-line drug selection (dosage)	Treatment duration
**Meningitis in neonates, causative agent not specified or unknown**		
NA (*WHO Model list of essential medicines for children*)^11^	First choice: Cefotaxime Second choice: Meropenem (dosage recommendation not provided)	NA
NA (*Pocket book of hospital care for children*)^12^	First choice: Ampicillin (dosage based on patient age and weight) and gentamicin (dosage based on patient age and weight). Alternatively: third-generation cephalosporin such as ceftriaxone (50 mg/kg every 12 hours if < 7 days of age and 75 mg/kg after 1 week) or cefotaxime (50 mg/kg every 12 hours if < 7 days or every 6–8 hours if > 7 days of age), and gentamicin for 3 weeks	3 weeks
Ghana^17^	Ceftriaxone (20–50 mg/kg once daily)	21 days
Malawi^21^	Benzylpenicillin (100 000 units/kg 6 hourly) plus gentamicin (2.5 mg/kg 8 hourly)	14–21 days
Nigeria^24^	Ceftriaxone (20–50 mg/kg daily, maximum dose: 50 mg/kg daily)	NA
Uganda^39^	Ampicillin IV (50–100 mg/kg every 8 hours for neonates < 7 days old or every 12 hours if > 7 days old) plus gentamicin (2.5 mg/kg every 12 hours)	21 days
Zambia^42^	Ceftriaxone (20–50 mg/kg daily as a single dose)	NA
Zimbabwe^43^	Fluconazole (6–12 mg/kg every 72 hours for neonates < 2 weeks old or every 48 hours for those 2 to 4 weeks old)	NA
**Meningitis in children, causative agent not specified or unknown^a^**		
NA (*WHO Model list of essential medicines for children*)^11^	First choice: Ceftriaxone Second choice: Amoxicillin, ampicillin, benzylpenicillin, chloramphenicol (for children > 2 years old)	NA
Liberia^20^	Ceftriaxone (50–100 mg/kg every 12 hours) or chloramphenicol (25 mg/kg/dose every 6 hours)	14 days
Malawi^21^	Benzylpenicillin (100 000 units/kg 6 hourly) plus chloramphenicol (25 mg/kg 8 hourly) or ceftriaxone (100 mg/kg every 24 hours)	7 days
Seychelles^26^	Benzylpenicillin (300 mg for infants < 1 year, 600 mg for 1- to 9-year-olds and 1 200 mg for children > 10 years)	NA
Uganda^39^	Ceftriaxone (100 mg/kg daily dose)	10–14 days
United Republic of Tanzania^40^	Ampicillin (50–100 mg/kg 6 hourly) or chloramphenicol (50 mg/kg 6 hourly)	10 days
**Meningitis caused by *Streptococcus pneumoniae* in paediatric patients^a^**		
Eswatini^13^	Benzylpenicillin (100 000 IU/kg per dose) or ceftriaxone (50–100 mg/kg in 1–2 divided doses)	10–14 days
Gambia^16^	Benzylpenicillin (dosage recommendation not provided)	10–14 days
Liberia^20^	Benzylpenicillin (100 000 IU/kg/dose every 4 hours) or ceftriaxone (50–100 mg/kg/dose once/twice a day)	10–14 days
South Africa^29^	Ceftriaxone (50 mg/kg/dose 12 hourly)	10 days
Uganda^39^	Benzylpenicillin (100 000 IU/kg per dose) or ceftriaxone (100 mg/kg daily dose)	10–14 days or up to 21 days in severe cases

IU: international unit; NA: not available; WHO: World Health Organization.

^a^ Excluding neonates.

Note: Antimicrobial treatment recommendations are summarized from selected standard treatment guidelines to illustrate the range of recommendations; additional standard treatment guidelines set forth recommendations and guidance but are not included here for brevity. Treatment recommendations were edited for grammar, clarity and/or length.

The variation between guidelines was less when the causative microorganism of the infection or syndrome was specified. For example, five guidelines provided recommendations for the treatment of acute meningitis in paediatric patients (excluding neonates) caused by *Streptococcus pneumoniae*. All five guidelines recommended monotherapy with either benzylpenicillin or ceftriaxone for 10 to 14 days; dosage recommendations were similar across the four guidelines that provided such information (Table 3).

Of the 14 countries that had national guidelines for the treatment of meningitis, only six (43%) included guidance or instructions for obtaining cultures and antimicrobial susceptibility testing or tailoring drug selection or duration accordingly (available in data repository).^44^ For example, the *Standard treatment guidelines and essential medicines list for South Africa, hospital level paediatrics* provide recommendations for empirical treatment of meningitis and instruct users to “Adjust antimicrobial therapy according to culture and sensitivity” and to “Re-assess antimicrobial therapy when blood and CSF [cerebrospinal fluid] culture and sensitivity results become available, or when improvement is not evident within 72–96 hours.”^29^ *The Gambia standard drug treatment guide* provides a recommendation to treat paediatric patients older than 2 months of age and presenting with meningitis with a combination therapy of benzylpenicillin plus chloramphenicol.^16^ Alongside the antimicrobial treatment recommendation, there is also guidance to “Continue until culture result is known, after which use a single drug therapy with chloramphenicol, benzylpenicillin or ampicillin.”^16^

#### Adult population

There were discrepancies among guidelines regarding the use of antimicrobials for the treatment of certain conditions for adult population. For example, guidelines from eight countries described recommendations for the treatment of acute bronchitis. Of those, three recommended treatments with various antimicrobial therapy regimens including 250–500 mg of amoxicillin for 5 days, 500 mg of amoxicillin every 8 hours for 7 days, 200 mg of doxycycline on the first day of treatment followed by 100 mg once a day for 5 days, and 500 mg of amoxicillin/clavulanic acid every 12 hours for 7 days (Table 4). Four other guidelines stated that antimicrobial use was not indicated for the treatment of acute bronchitis while a fifth guideline stated that antibiotics were not indicated for uncomplicated bronchitis but for purulent bronchitis, treatment with amoxicillin at a dosage of 500 mg every 8 hours for 4 or more days was indicated. All four guidelines for the treatment of chronic bronchitis indicated treatment with an antimicrobial agent. The WHO model list^10^ did not provide clinical guidance for the treatment of acute or chronic bronchitis in adults.

**Table 4:** Summary of treatment recommendations from included African standard treatment guidelines for acute and chronic bronchitis for adult patient populations, 2001–2019

Country	Recommended first-line drug selection (dosage)^a^	Treatment duration
**Acute bronchitis**		
Eswatini^13^	Antibiotics are not indicated for uncomplicated bronchitis, but if purulent treat with amoxicillin (500 mg every 8 hours)	4 or more days
Ghana^17^	Amoxicillin (500 mg 8 hourly) or amoxicillin/clavulanic acid (one 500/125 tablet 12 hourly); double the dose if severe	7 days
Kenya^18^	Amoxicillin (250–500 mg three times a day) or tetracycline	5 days
Malawi^21^	Amoxycillin (500 mg three times/day) or doxycycline (200 mg on first day followed by 100 mg once/day)	5 days
Nigeria^24^	Not required unless clear evidence of primary bacterial etiology or secondary bacterial infection	Not applicable
Seychelles^26^	Consider 7-day delayed antibiotic with symptomatic advice/leaflet. Care should be taken to exclude a differential diagnosis of pneumonia. Antibiotics are not indicated in people who are otherwise well. Routine follow-up is not necessary. However, patients should be advised to seek advice if their condition deteriorates significantly, or symptoms persist for longer than 3 weeks. Consider antibiotics for those with pre-existing conditions that impair their ability to fight infection or are likely to deteriorate with acute bronchitis	Not applicable
United Republic of Tanzania^40^	There is no benefit from antibiotic use. Pertussis is the only indication for antibacterial agents in the treatment of acute bronchitis	Not applicable
Zimbabwe^43^	No antibiotics required	Not applicable
**Chronic bronchitis**		
Eswatini^13^	If there is infection, treat with amoxicillin (500 mg every 8 hours)	Not specified
Gambia^16^	For secondary infection: amoxicillin (500 mg 8 hourly) or erythromycin (500 mg 6 hourly) or azithromycin (500 mg daily)	Amoxicillin, erythromycin: 7 days Azithromycin: 3 days
Liberia^20^	Co-trimoxazole (960 mg every 12 hours) or amoxicillin (500 mg over 8 hours)	5 days
Zimbabwe^43^	Treat with antibiotics (amoxicillin 500 mg three times/day) or doxycycline (100 mg once/day for 7 days) if sputum colour has changed to purulent, if there is fever or new chest X-ray infiltrates	7 days

^a^ We summarized treatment recommendations from the standard treatment guidelines and edited for grammar, clarity and/or length.

## Discussion

The misuse and overuse of antimicrobials in the human health sector is a major driver of antimicrobial resistance globally. Per capita antimicrobial consumption in Africa and other low- and middle-income countries is rising at a faster rate than high-income countries.^45^ In part, this consumption pattern may be due to a lack of clear guidance for the use of antimicrobials in the clinical setting, improved access to antimicrobials, and non-prescription use of antimicrobials.^45^ Here, we found that only one third of AU Member States had any relevant standardized treatment guidelines for the treatment of common bacterial infections or syndromes. No guidelines stated that they were based on local disease burden or resistance profiles; one explanation for this finding may be the lack of national laboratory and surveillance capacities leading to gaps in the local evidence base. Only a small number of guidelines cited published literature or other clinical evidence supporting the rationale for certain drug, dosage and duration recommendations. Few guidelines incorporated antimicrobial stewardship principles, culture or antimicrobial susceptibility testing results into treatment recommendations.

Only about half of the bacterial infections and/or syndromes covered in existing standardized treatment guidelines from AU Member States across adult and paediatric patient populations were addressed in WHO model lists^10^^,^^11^ and less than a third were included in the *Pocket book of hospital care for children*.^12^ This finding indicates a potential discrepancy between disease burden and priorities at the international and regional contexts and reinforces the need for context-specific standardized treatment guidelines to guide the appropriate clinical treatment of these infections and clinical syndromes. Finally, the recommended drug selection, dosage and duration of therapy varied across the guidelines, indicating a lack of clear, consensus clinical guidance and potential for the misuse of antimicrobials.

Clinical guidelines that provide explicit recommendations for the treatment of infectious diseases and aid in clinical decision-making have been shown to reduce inappropriate antimicrobial prescribing as well as improve the quality of care.^46^^,^^47^ High quality treatment guidelines should be based on a rigorous multidisciplinary evaluation of all available scientific evidence about treatment effectiveness and local disease and antimicrobial resistance burden.^7^^,^^48^^,^^49^ Moreover, as antimicrobial resistance increasingly poses a public health threat, guidelines must also encourage clinicians to obtain cultures, identify the causative microorganism and conduct antimicrobial susceptibility testing before or during therapeutic intervention. 

This study had several limitations. First, we did not include guidelines developed by nongovernmental public or private institutions, professional organizations or individual health facilities in this review, which may have limited the number of total countries represented in this study. Moreover, guidelines developed outside of the national government infrastructure may represent an important source for information guiding clinical decision-making and antimicrobial prescribing practices at the facility, national and regional levels. While we made every effort to identify all existing guidelines for each eligible country, some guidelines used in clinical practice may not be publicly available or easily searchable online and may have been omitted from inclusion. In the future, all efforts should be exhausted to ensure that all applicable guidelines are identified and considered for inclusion; maintenance of a guidelines database may also be useful. In addition, this study does not account for the awareness of, utilization of and/or adherence to standardized treatment guidelines at the local or national levels nor other factors that may influence feasibility of using guidelines, such as availability and cost of recommended antimicrobials. As such, antimicrobial treatment regimens employed in clinical practice may vary widely from those recommended in any standardized treatment guidelines.

In addition to the suboptimal treatment guidelines, the high antimicrobial consumption rates in the African Region may reflect the higher burden of infectious diseases, the high prevalence of substandard pharmaceutical agents, the lack of access to health-care services and lack of regulation around prescription-only use of antimicrobials. These factors lead to high rates of self-medication and non-prescription antimicrobials sales and consumption.^50^ Therefore, while the development and implementation of standardized treatment guidelines offer an opportunity to reduce the misuse and overuse of antimicrobials in the human health sector, other antimicrobial stewardship interventions that address other drivers of antimicrobial overuse and misuse and the emergence and spread of antimicrobial resistance more broadly must also be implemented.

In conclusion, AU Member States need to develop standardized treatment guidelines that meet internationally accepted standards for methods and are based on locally derived clinical evidence, disease burden and resistance profiles. To achieve this, countries must also increase their capacity for evidence building, including antimicrobial resistance and infectious disease surveillance. Furthermore, many countries across the continent lack adequate access to clean water and sanitation facilities, and have vaccination coverage below recommended levels. In addition to ensuring antimicrobial treatment is appropriate, preventing infections where possible is essential to reduce antimicrobial use, slow the emergence of resistance and ultimately improve health outcomes and save lives.

## References

[1] Tadesse BT, Ashley EA, Ongarello S, Havumaki J, Wijegoonewardena M, González IJ, et al. Antimicrobial resistance in Africa: a systematic review. BMC Infect Dis. 2017 Sep 11;17(1):616. 10.1186/s12879-017-2713-1 28893183 PMC5594539

[2] Elton L, Thomason MJ, Tembo J, Velavan TP, Pallerla SR, Arruda LB, et al.; the PANDORA-ID-NET consortium. Antimicrobial resistance preparedness in sub-Saharan African countries. Antimicrob Resist Infect Control. 2020 Aug 28;9(1):145. 10.1186/s13756-020-00800-y 32859252 PMC7456056

[3] Laxminarayan R, Matsoso P, Pant S, Brower C, Røttingen JA, Klugman K, et al. Access to effective antimicrobials: a worldwide challenge. Lancet. 2016 Jan 9;387(10014):168–75. 10.1016/S0140-6736(15)00474-2 26603918

[4] Essack SY, Desta AT, Abotsi RE, Agoba EE. Antimicrobial resistance in the WHO African region: current status and roadmap for action. J Public Health (Oxf). 2017 Mar 1;39(1):8–13. 10.1093/pubmed/fdw015 26944074 PMC5939661

[5] Africa CDC Framework for Antimicrobial Resistance 2018–2023. Addis Ababa: Africa Centre for Disease Control; 2018. Available from: https://africacdc.org/download/africa-cdc-framework-for-antimicrobial-resistance/ [cited 2021 June 18].

[6] CDC. Core elements of hospital antibiotic stewardship programs. Atlanta: US Department of Health and Human Services, Centers for Disease Control and Prevention; 2019. Available from: https://www.cdc.gov/antibiotic-use/core-elements/hospital.html [cited 2021 June 18].

[7] Antibiotic stewardship statement for antibiotic guidelines – recommendations of the Healthcare Infection Control Practices Advisory Committee. Atlanta: Healthcare Infection Control Practices Advisory Committee; 2017. Available from: https://www.cdc.gov/hicpac/recommendations/antibiotic-stewardship-statement.html [cited 2021 June 18].

[8] Member State profiles. Addis Ababa: African Union; 2020. Available from: https://au.int/memberstates [cited 2021 June 18].

[9] Kavanagh BP. The GRADE system for rating clinical guidelines. PLoS Med. 2009 Sep;6(9):e1000094. 10.1371/journal.pmed.1000094 19753107 PMC2735782

[10] WHO model list of essential medicines, 21st List, 2019. Geneva: World Health Organization; 2019. Available from: https://www.who.int/groups/expert-committee-on-selection-and-use-of-essential-medicines/essential-medicines-lists [cited 2021 Nov 15].

[11] WHO model list of essential medicines for children, 7th List, 2019. Geneva: World Health Organization; 2019. Available from: https://www.who.int/publications/i/item/WHOMVPEMPIAU201907 [cited 2021 June 18].

[12] Pocket book of hospital care for children: guidelines for the management of common childhood illnesses. 2nd ed. Geneva: World Health Organization; 2013. 24006557

[13] Standard treatment guidelines and essential medicines list of common medical conditions in the Kingdom of Swaziland. 1st ed. Manzini: Kingdom of Swaziland Ministry of Health; 2012. Available from: http://www.gov.sz/images/standard%20treatment%20guidelines.pdf [cited 2021 Nov 15].

[14] Guideline on cholera outbreak management. Addis Ababa: Ethiopian Health and Nutrition Research Institute; 2011. Available from: http://repository.iifphc.org/bitstream/handle/123456789/373/national-cholera-guideline.pdf?sequence=1&isAllowed=y [cited 2021 Nov 1].

[15] National guidelines for the management of sexually transmitted infections using syndromic approach. CITY: Federal Democratic Republic of Ethiopia Ministry of Health; 2015. Available from: https://www.medbox.org/pdf/5e148832db60a2044c2d3463 [cited 2021 Nov 15].

[16] The Gambia standard drug treatment guides. Banjul: Department of State for Health and Social Welfare; 2001. Available from: https://www.moh.gov.gm/wp-content/uploads/2021/05/Standard_Drug_Treatment_Guidelines.pdf [cited 2021 Nov 15].

[17] Standard treatment guidelines. 6th ed. Accra: Ministry of Health Ghana; 2010.

[18] Clinical guidelines for the management and referral of common conditions at level 4-6: hospitals. Nairobi: Ministry of Medical Services and Ministry of Public Health and Sanitation; 2009.

[19] Guidelines on cholera control. Nairobi: Ministry of Health; 2002. Available from: http://guidelines.health.go.ke/#/category/11/43/meta [cited 2021 Nov 15].

[20] 2nd edition national standard therapeutic guidelines and essential medicines list Liberia, 2017. Monrovia: Republic of Liberia Ministry of Health; 2017. Available from: https://moh.gov.lr/wp-content/uploads/Liberia-NSTG-EML-2nd-Edition-2017.pdf [cited 2021 Nov 1].

[21] Malawi standard treatment guidelines (MSTG). 5th edition. Lilongwe: Ministry of Health; 2015. Available from: https://www.medbox.org/document/malawi-standard-treatment-guidelines-mstg-5th-edition#GO [cited 2021 Nov 15].

[22] [Directives de prise en charge de l’enfant malade de moins de cinq ans]. Rabat: Ministere de la Sante; 2016. French.

[23] Namibia standard treatment guidelines. 1st ed. Windhoek: Republic of Namibia Ministry of Health and Social Services; 2011. Available from: http://www.man.com.na/files/news/1501069447namibia-standard-treatment-guidelines.pdf [2021 Nov 15].

[24] Nigeria standard treatment guidelines. 2nd ed. Abuja: Federal Ministry of Health; 2016. Available from: https://www.medbox.org/document/nigeria-standard-treatment-guidelines#GO [cited 2021 Oct 1].

[25] Internal medicine clinical treatment guidelines. Kigali: Republic of Rwanda Ministry of Health; 2012. Available from: https://www.medbox.org/pdf/5e148832db60a2044c2d1f64 [cited 2021 Nov 15].

[26] Antimicrobial guidelines for management of infections in hospitals. Victoria: Ministry of Health Care Agency; 2018.

[27] Guidelines for antibiotic prescribing in the primary health care services. Victoria: Health Care Agency; 2017.

[28] Somali treatment guidelines in line with the essential package of health services primary health unit STGs. Mogadishu: Somali Health Authorities and World Health Organization; 2015. Available from: https://reliefweb.int/sites/reliefweb.int/files/resources/1._primary_health_unit_stgs_-_federal_government_of_somalia.pdf [cited 2021 Nov 15].

[29] Standard treatment guidelines and essential medicines list for South Africa: hospital level paediatrics, 2017 edition. Pretoria: The National Department of Health Republic of South Africa; 2017. Available from: http://www.kznhealth.gov.za/pharmacy/paediatric-stgs-eml_4thed2017.pdf [cited 2021 Nov 15].

[30] Standard treatment guidelines and essential medicines list for South Africa: hospital level adults. Pretoria: National Department of Health; 2015. Available from: https://www.sapc.za.org/Media/Default/Documents/STG%20hospital%20level%20adult%202019_v2.0.pdf [cited 2021 Nov 15].

[31] Standard treatment guidelines and essential medicines list for South Africa: primary healthcare level. Pretoria: National Department of Health; 2018. Available from: https://extranet.who.int/ncdccs/Data/ZAF_D1aia_STG%20and%20EML%20PHC%202018.pdf [cited 2021 Nov 15].

[32] Guidelines on leprosy control in South Africa. Pretoria: Department of Health; 2011. Available from https://www.nicd.ac.za/assets/files/Leprosy%20Control%20in%20SA%202011.pdf [cited 2021 Nov 15].

[33] Listeriosis: clinical recommendations for diagnosis and treatment. Johannesburg: National Institute for Communicable Diseases; 2017. Available from: https://www.nicd.ac.za/wp-content/uploads/2017/12/Listeriosis_Clinical_Guidelines.pdf [cited 2021 Nov 1].

[34] Sudan national standard treatment guidelines. Khartoum: Federal Ministry of Health; 2014. Available from: http://www.sho.gov.sd/controller/dwn_hub_files.php?id=915 [cited 2021 Nov 15].

[35] [Direction de la pharmacie et des medicaments- antibiotherapie des infections osteo-articulaires aigues communautaires a pyogenes]. Tunis: Ministere de la Sante Publique; 2006. French.

[36] Redjeb CSBEN, Bouzouaia N, De G, Chaabane TBEN, Sfax SBENH, Sfax MBENJ, et al. [Antibiotherapie des pyelonephrites aigues communautaires de l’adulte]. Tunis: Ministère de la santé publique; 2016. French.

[37] [L’antibiotherapie dans les infections respiratoires basses acquises de l’adulte traitee en ville]. Tunis: Ministere de la Sante. French.

[38] Prevention and control of cholera. Kampala: The Republic of Uganda Ministry of Health; 2017. Available from: http://library.health.go.ug/download/file/fid/1696 [cited 2021 Nov 15].

[39] Uganda clinical guidelines 2016. Kampala: The Republic of Uganda Ministry of Health; 2016. Available from: http://library.health.go.ug/publications/guidelines/uganda-clinical-guidelines-2016 [cited 2021 Nov 15].

[40] Standard treatment guidelines (STG) and the national essential medicines list (NEMLIT) for mainland Tanzania. 3rd ed. Dodoma: The Ministry of Health Community Development Gender Elderly and Children; 2007. Available from: https://www.who.int/selection_medicines/country_lists/tza_stg_neml_2007.pdf [cited 2021 Nov 15].

[41] Standard treatment guidelines, essential medicines list and essential laboratory supplies list for Zambia. Lusaka: Republic of Zambia Ministry of Health; 2013. Available from: https://www.moh.gov.zm/docs/final_standard_treatment_guidelines_booklet_04.pdf [cited 2021 Nov 15].

[42] Essential newborn care guidelines. Lusaka: Ministry of Community Development Mother and Child Health; 2014. Available from: https://www.afro.who.int/publications/essential-newborn-care-guidelines-2014 [cited 2021 Nov 15].

[43] 7th essential medicines list and standard treatment guidelines for Zimbabwe. Harare: The National Medicine and Therapeutics Policy Advisory Committee; 2015. Available from: http://www.mdpcz.co.zw/wp-content/uploads/2018/10/EDLIZ.pdf [cited 2021 Nov 15].

[44] Craig J, Hiban K, Frost I, Kapoor G, Alimi Y, Varma JK. Antimicrobial treatment recommendations for common bacterial infections and syndromes from national/standard treatment guidelines in African Union member states. Comparison of national antimicrobial treatment guidelines, African Union. Supplemental information [data repository]. Geneva: CERN; 2021. 10.5281/zenodo.5682177 10.5281/zenodo.5682177

[45] Klein EY, Van Boeckel TP, Martinez EM, Pant S, Gandra S, Levin SA, et al. Global increase and geographic convergence in antibiotic consumption between 2000 and 2015. Proc Natl Acad Sci USA. 2018 04 10;115(15):E3463–70. 10.1073/pnas.1717295115 29581252 PMC5899442

[46] Versporten A, Zarb P, Caniaux I, Gros MF, Drapier N, Miller M, et al.; Global-PPS network. Antimicrobial consumption and resistance in adult hospital inpatients in 53 countries: results of an internet-based global point prevalence survey. Lancet Glob Health. 2018 Jun;6(6):e619–29. 10.1016/S2214-109X(18)30186-4 29681513

[47] Fernández González F, Detrés J, Torrellas P, Balleste CR. Comparison of the appropriate use of antibiotics based on clinical guidelines between physicians in-training versus practicing physicians. Bol Asoc Med P R. 2013;105(3):21–4. 24282916

[48] de With K, Allerberger F, Amann S, Apfalter P, Brodt HR, Eckmanns T, et al. Strategies to enhance rational use of antibiotics in hospital: a guideline by the German Society for Infectious Diseases. Infection. 2016 Jun;44(3):395–439. 10.1007/s15010-016-0885-z 27066980 PMC4889644

[49] Kredo T, Bernhardsson S, Machingaidze S, Young T, Louw Q, Ochodo E, et al. Guide to clinical practice guidelines: the current state of play. Int J Qual Health Care. 2016 Feb;28(1):122–8. 10.1093/intqhc/mzv115 26796486 PMC4767049

[50] Grijalva CG, Nuorti JP, Griffin MR. Antibiotic prescription rates for acute respiratory tract infections in US ambulatory settings. JAMA. 2009 Aug 19;302(7):758–66. 10.1001/jama.2009.1163 19690308 PMC4818952

